# Rewiring Meiosis for Crop Improvement

**DOI:** 10.3389/fpls.2021.708948

**Published:** 2021-07-19

**Authors:** Pallas Kuo, Olivier Da Ines, Christophe Lambing

**Affiliations:** ^1^Department of Plant Sciences, University of Cambridge, Cambridge, United Kingdom; ^2^Institut Génétique Reproduction et Développement (iGReD), Université Clermont Auvergne, UMR 6293 CNRS, U1103 INSERM, Clermont-Ferrand, France

**Keywords:** meiosis, meiotic recombination, centromere, telomere, plant, chromatin, epigenetic, ploidy

## Abstract

Meiosis is a specialized cell division that contributes to halve the genome content and reshuffle allelic combinations between generations in sexually reproducing eukaryotes. During meiosis, a large number of programmed DNA double-strand breaks (DSBs) are formed throughout the genome. Repair of meiotic DSBs facilitates the pairing of homologs and forms crossovers which are the reciprocal exchange of genetic information between chromosomes. Meiotic recombination also influences centromere organization and is essential for proper chromosome segregation. Accordingly, meiotic recombination drives genome evolution and is a powerful tool for breeders to create new varieties important to food security. Modifying meiotic recombination has the potential to accelerate plant breeding but it can also have detrimental effects on plant performance by breaking beneficial genetic linkages. Therefore, it is essential to gain a better understanding of these processes in order to develop novel strategies to facilitate plant breeding. Recent progress in targeted recombination technologies, chromosome engineering, and an increasing knowledge in the control of meiotic chromosome segregation has significantly increased our ability to manipulate meiosis. In this review, we summarize the latest findings and technologies on meiosis in plants. We also highlight recent attempts and future directions to manipulate crossover events and control the meiotic division process in a breeding perspective.

## Chromatin and Recombination in Meiosis

### Meiotic Recombination

Meiosis is a specialized cell division taking place in sexually reproducing organisms during which a cell undergoes two rounds of chromosome segregation to form four daughter cells of halved ploidy. Each daughter cell contains a set of chromosomes with varying genetic contents to the others due to genetic exchanges and random assortment of homologous chromosomes and sister chromatids. The first meiotic segregation faces a unique situation whereby chromosomes undergo recombination events leading to reciprocal exchanges between homologs, also called crossovers (COs; Mercier et al., 2015). COs are important to create novel genetic diversity, and this natural process is utilized during breeding strategies to break the linkage between genes, facilitating the removal of unfavorable genetic elements or improving the mapping of quantitative trait locus (Mercier et al., 2015).

Meiotic recombination initiates with the formation of programmed DNA double-strand breaks (DSBs) induced by a topoisomerase-like complex related to the archaeal TopoVI DNA topoisomerase (Topo VI). Topo VI is an heterotetrameric enzymatic complex comprising two A and two B subunits and catalyzing DNA strand breakages (Bergerat et al., 1997). In meiosis, SPO11 and MTOPVIB form a complex with topoisomerase-like activity to create a DSB onto which SPO11 remains covalently bound to the DSB end *via* a phosphotyrosyl bond (Robert et al., 2016; Vrielynck et al., 2016). SPO11 forms meiotic DSBs as a homodimer in animals and fungi, and as a SPO11-1/SPO11-2 heterodimer in plants (Mercier et al., 2015). Studies of SPO11 proteins between plant species reveal that the number of orthologs varies greatly (Sprink and Hartung, 2014; Da Ines et al., 2020). In *Arabidopsis thaliana*, three SPO11 paralogs are identified but only SPO11-1 and SPO11-2 are involved in meiotic DSB formation (Hartung et al., 2007). Rice has five SPO11 paralogs and only SPO11-1 and SPO11-2 have a confirmed role in meiosis while loss of *spo11-4* has no meiotic defects (Yu et al., 2010; Fayos et al., 2020). The high number of SPO11 paralogs in plants makes genetic engineering to control meiotic recombination more challenging. However, SPO11 orthologs are sufficiently conserved between plant species as to complement each other’s loss of function mutations. For instance, expression of bread wheat SPO11-2 restores fertility in Arabidopsis *spo11-2* (Benyahya et al., 2020; Da Ines et al., 2020) while expression of bread wheat SPO11-1-5D restores fertility in both rice and Arabidopsis *spo11-1* (Da Ines et al., 2020). Additional proteins are required for DSB formation and appear conserved between plants (Jing et al., 2019). For example, Arabidopsis PRD1 (De Muyt et al., 2007), PRD2 and PRD3 (De Muyt et al., 2009), and DFO (Zhang et al., 2012) are all essential for the formations of meiotic DSB. Similarly, rice *prd1* (Shi et al., 2021), *mtopVIb* (Fu et al., 2016; Xue et al., 2016) and *prd3/pair1* (Nonomura et al., 2004), and maize *mtopVIb* (Jing et al., 2020) are defective in DSB formation.

Cytological studies using DNA damage markers, such as γ-H2AX and RAD51, revealed the formation of a large number of DSBs in early meiosis. It is estimated that between 150 and 200 meiotic DSBs are formed in Arabidopsis and between ~200 and 2,000 in crops with larger genome (Ferdous et al., 2012; Higgins et al., 2012; Sidhu et al., 2015; Gardiner et al., 2019; Benyahya et al., 2020). DSBs are formed on the chromatin which is organized in arrays of loops anchored to a proteinaceous linear structure called the chromosome axis (Zickler and Kleckner, 1999; Kleckner, 2006). In plants, components of the chromosome axis include meiotic cohesin REC8 (Chelysheva et al., 2005; Golubovskaya et al., 2006), HORMA-domain-containing proteins ASY1/PAIR2 (Armstrong et al., 2002; Nonomura et al., 2006), and coiled-coil proteins ASY3/PAIR3/DSY2 (Wang et al., 2011; Ferdous et al., 2012; Lee et al., 2015) and ASY4 (Chambon et al., 2018; Osman et al., 2018). The axis proteins ASY3/DSY2/Red1 are essential for DSB formation (Panizza et al., 2011; Ferdous et al., 2012; Lee et al., 2015), and chromosome axis length covaries with the number of DSB markers on a per-nucleus basis in Arabidopsis and budding yeast, highlighting the important regulatory functions of the axis on recombination initiation (Wang et al., 2019b; Lambing et al., 2020b).

Following DSB formation, DSB ends are resected by the MRN/COM1 complex to form 3' overhang single-stranded DNA (ssDNA) onto which RPA, RAD51, and DMC1 are recruited to form nucleoprotein filaments (Mercier et al., 2015). Multiple strand invasions of the chromosome filaments favor homologous chromosome alignment and are critical for chromosome pairing in most species (Cloud et al., 2012; Hong et al., 2013). Successful pairing leads to installation of a tripartite structure called the synaptonemal complex (SC) which consists of a transverse filament formed with ZYP1 and connecting the two homologous axes (Mercier et al., 2015). The SC initiates at recombination sites (Zhang et al., 2014a; Lambing et al., 2015), and several lines of evidence suggest that SC components regulate CO formation (Higgins et al., 2005; Barakate et al., 2014; Chen et al., 2015; Voelkel-Meiman et al., 2015; Capilla-Perez et al., 2021; France et al., 2021). Meiotic DSB repair results in a CO or a non-CO, with a possibility of gene conversion in either case (Berchowitz and Copenhaver, 2010). Gene conversions are short unidirectional exchanges (few hundreds base pairs) of genetic information between chromosomes. Gene conversion events are rare, and the control over gene conversion is not well understood. The frequency of gene conversion per meiosis on a given locus is estimated between ~10^−4^ and 10^−6^ (Sun et al., 2012; Drouaud et al., 2013; Wijnker et al., 2013). Gene conversion frequency is associated with MSH4, a protein required for CO formation (Drouaud et al., 2013), and can be detected on the heterochromatin regions where COs are repressed (Shi et al., 2010).

How a DSB’s fate is determined is still not fully understood, but it is thought that pro- and anti-CO pathways influence the repair outcome at DSB sites (Mercier et al., 2015). For example, a set of proteins collectively named “ZMM” (SHOC1, PTD, HEI10, ZIP4, MER3, MSH4, and MSH5 in Arabidopsis) stabilizes inter-homolog joint molecules and promotes CO formation (Mercier et al., 2015). In contrast, anti-CO proteins, such as FANCM, BLM/RECQ4, TOP3*α*, FIGL1, disengage joint molecules *via* helicase or topoisomerase activities, and repress CO formation (Crismani et al., 2012; Mercier et al., 2015; Seguela-Arnaud et al., 2015). During meiotic DSB repair, the ssDNA ends elongate *via* DNA synthesis using the homologous chromosome as a template. If heterozygosity is shared between the homologous template and the ssDNA end, disengagement of this ssDNA and repair by an anti-CO pathway could lead to a non-CO associated with a gene conversion (Berchowitz and Copenhaver, 2010). Recent studies in budding yeast indicate that complex partner switches may be common during meiosis, creating chromatids with mosaic allelic patterns (Mcmahill et al., 2007; Marsolier-Kergoat et al., 2018).

Each bivalent chromosome must form at least one CO, termed an obligate CO, to form the physical link between chromosomes which is essential to ensure proper chromosome segregation in meiosis. In most species, CO formation is limited to 1–3 per chromosome pair (Jones and Franklin, 2006; Mercier et al., 2015). Several factors have been reported to contribute to these phenomena: CO homeostasis (Henderson and Keeney, 2004), CO interference (Kleckner et al., 2004), limited amount of pro-CO factors (Ziolkowski et al., 2017), and the presence of anti-CO factors (Crismani et al., 2012; Girard et al., 2015; Seguela-Arnaud et al., 2015). CO homeostasis is a phenomenon buffering changes in DSB number for the maintenance of total COs. In this context, an elevation or a decrease in DSBs does not impact CO number. While CO homeostasis is observed in budding yeast (Martini et al., 2006), it may be different in plants (Sidhu et al., 2015; Xue et al., 2018). In contrast, CO interference is a phenomenon resulting in the non-random distribution of COs whereby the formation of one CO inhibits the formation of additional COs in adjacent regions, thus preventing clustering of COs (Wang et al., 2015). Although factors involved in this phenomenon are unclear, it has been suggested that a combination of physical stresses generated from the expansion and contraction of chromatin compressing the chromosome axis during prophase I, combined with the diffusion of proteins along the axis, contribute to the establishment of an interfering signal (Wang et al., 2015; Zhang et al., 2018). In accordance with this model, components of the chromosome axis have been implicated in CO interference in budding yeast (Zhang et al., 2014b), *Caenorhabditis elegans* (Libuda et al., 2013; Zhang et al., 2018), and Arabidopsis (Lambing et al., 2020a; Capilla-Perez et al., 2021; France et al., 2021). However, the chromosome axis in itself may not be sufficient to impose CO interference since axis is formed in *asy1* and *zyp1* mutant lines in which interference is lost (Lambing et al., 2020a; Capilla-Perez et al., 2021; France et al., 2021).

### Chromatin and DSB Hotspots

DSBs are not randomly formed on the genome. Instead, they are enriched in nucleosome-depleted regions (Pan et al., 2011; Lam and Keeney, 2015; Choi et al., 2018). It appears that regions with high nucleosome occupancy prevent SPO11 accessibility and thus restrict DSB formation. DSB formation also takes place in the context of chromatin loops anchored to a chromosome axis. Counterintuitively, certain components of the DSB machinery are found associated with the chromosome axis while DSBs are located in the chromatin loops, away from the axial sites in budding yeast (Panizza et al., 2011; Stanzione et al., 2016). To reconcile the two observations, it was proposed that chromatin loops are tethered to the axis prior to DSB formation. In support of this model, Spp1, a PHD finger-domain protein, was found to interact with H3K4me3 modifications located on the chromatin loop, and with Mer2 protein, a component of the DSB machinery located on the axis in budding yeast (Acquaviva et al., 2013; Sommermeyer et al., 2013). This observation indicates a complex interaction between chromatin loop organization, epigenetics marks, and recombination. Interestingly, DSB hotspots are enriched at the 5' end of genes, and axis components are enriched at the 3' end of genes and are influenced by transcriptional activity in budding yeast (Pan et al., 2011; Lam and Keeney, 2015; Medhi et al., 2016). Arabidopsis DSB maps show enrichment of DSBs at the 5' and 3' end of genes, in regions of low nucleosome occupancy and with markers of open chromatin (e.g., H3K4me3). DSBs are correspondingly depleted in heterochromatic regions that are enriched in transposons, GC content, and DNA methylation (Choi et al., 2018). Consistent with budding yeast, ChIP-seq of Arabidopsis axis protein revealed that REC8 and DSBs occupy distinct sites. REC8 also shows a preferential polarization toward the end of genes that is influenced by transcriptional activity (Lambing et al., 2020b). Comparing genome-wide Arabidopsis axis and DSB profiles revealed no correlation between the enrichment of SPO11-1-oligos and REC8 or ASY1 over genes, indicating that although the chromosome axis is important for DSB formation, the amount of axis protein does not specify the frequency of DSBs locally (Lambing et al., 2020a,b). Additional factors likely influence the local frequency of DSB formation.

### Influence of Heterochromatin and Centromeres on Meiosis

Although COs are suppressed over the heterochromatin, a substantial number of DSBs has been detected over the pericentromeric heterochromatic regions, including at transposons, in Arabidopsis (Choi et al., 2018; Underwood et al., 2018) and maize (He et al., 2017). The maize genome is ~85% transposons, and comparative analysis shows that DSBs are distributed along the entire chromosomes without specific polarization, while COs are skewed toward the end of the chromosomes (He et al., 2017). Few COs were reported in the heterochromatic knob regions in maize but at much lower frequency than its DSB frequency (Stack et al., 2017). Thus, an interesting possibility is that recombination may not be fully suppressed on the heterochromatin and centromeres but rather channeled to favor non-CO outcomes, such as inter-sister repair. Indeed, gene conversions were detected in maize centromeric regions (Shi et al., 2010).

CO suppression over the centromeric heterochromatin is widely conserved (Ellermeier et al., 2010; Li et al., 2015; Phillips et al., 2015). The molecular mechanisms allowing this suppression are not clear, but appear instrumental since centromeric COs have been associated with increased rates of mis-segregation and aneuploidy in multiple species (Fernandes et al., 2019). On the other hand, understanding suppression of CO at or close to the centromeres is of particular importance for breeding, given that lack of meiotic CO in pericentromeric regions is a major bottleneck in varietal development of crop plants.

Centromeres are the sites of kinetochore assembly which enable microtubule fiber attachment and thus faithful segregation of chromosomes during mitotic and meiotic division. The structure and organization of the centromeres vary considerably between species with centromeres occupying a short sequence, a region or even the entire chromosome (Steiner and Henikoff, 2015; Talbert and Henikoff, 2020). Point centromeres are typical in budding yeast (Prosee et al., 2020) while *C. elegans* and some plants display holocentric chromosomes where the whole chromosome acts as a centromere (Melters et al., 2012). Holocentric chromosomes impose a specific problem to meiosis and how meiosis is remodeled in holocentric plants is being extensively investigated (Marques and Pedrosa-Harand, 2016). Most plants and mammals, however, exhibit regional centromeres. In plants, regional centromeres are largely composed of centromeric satellite repeats and centromeric retrotransposon arrays that can be several megabases long (Lamb et al., 2007; Ma et al., 2007; Fernandes et al., 2019). Yet, a centromere is generally not defined by a specific DNA sequence but rather by the presence of the specific histone H3 variant CenH3 (mammalian CENP-A), which acts as a particular epigenetic mark that establishes functional centromeres. CenH3 is present at all functional centromeres independently of their DNA sequence, and this epigenetic specification of centromere identity is broadly conserved in eukaryotes (Steiner and Henikoff, 2015; Fernandes et al., 2019; Talbert and Henikoff, 2020).

How centromeres function during meiosis in plants is still poorly understood but a number of studies have described the essential role of early centromere associations in homologous chromosome recognition, pairing, and subsequent synapsis during meiosis (reviewed in Da Ines and White, 2015; Sepsi and Schwarzacher, 2020). Remarkably, early centromere associations seem not directly mediated by DSB formation and recombination but rather by local chromatin homology, although stabilization of centromere pairing appears to be partially dependent on recombination initiation (Da Ines et al., 2012; Da Ines and White, 2015; Sepsi and Schwarzacher, 2020). Centromere association requires active centromeres and the presence of functional CENH3 variants (Zhang et al., 2013). Thus, despite high-DNA sequence homology, initial centromere interactions are driven by specific chromatin structure and centromeric proteins. In particular, early centromere associations are strongly dependent on the REC8 cohesin enrichment as well as DNA repeats organization at centromeres. In wheat, recent work has revealed that centromere satellite organization has diverged in the different wheat sub-genomes and these rearrangements of CENH3 nucleosomes likely influence centromere interaction and further homologous chromosome pairing (Su et al., 2019).

It is possible that early centromere association may impede access of the recombination machinery and thereby may play a key role in suppressing CO at centromeres. This is supported by the recent demonstration that REC8 enrichment is strongly correlated with suppression of meiotic DSBs and crossovers in Arabidopsis (Lambing et al., 2020b). Given that REC8 cohesin protein is highly enriched at centromeric sites from early meiosis I up to meiosis II and that centromere coupling and pairing also require the presence of REC8 (Cai et al., 2003; Zhang et al., 2013), it is conceivable that early centromere associations are intricately linked to suppression of recombination at centromeres.

## Engineering Meiotic Recombination

### Increasing Meiotic Recombination Genome Wide

In most plants, only few COs are formed on each chromosome per meiosis and this phenomenon limits the potential to create novel genetic diversity (Mercier et al., 2015). This is caused in part by a limited amount of pro-CO factors, the repressive activity of anti-CO factors and the action of CO interference. The majority of COs is formed by the ZMM pathway. Among actors of this pathway, the E3 ligase HEI10 is dosage-dependent for recombination, with an increase in *HEI10* expression being sufficient to increase the total genetic map length by 2-fold in hybrid Arabidopsis, but with limited effect on the CO rate over the heterochromatic regions (Ziolkowski et al., 2017). Overexpression of *HEI10* in Arabidopsis is also found to decrease CO interference although it is unclear how HEI10 impacts this process (Serra et al., 2018). The regulation of HEI10 dosage is a promising avenue to increase CO number in crops by stabilizing the recombination events maturing into class I COs and reducing the strength of CO interference. Recent studies identified protein phosphatase X1 and ZYP1/ZEP1 as additional factors limiting class I CO formation suggesting that other strategies may be possible to increase class I CO rate (Wang et al., 2010, 2015; Capilla-Perez et al., 2021; France et al., 2021; Nageswaran et al., 2021).

Several anti-CO factors affecting class II COs have been identified with non-functional redundancy (Mercier et al., 2015; Wang and Copenhaver, 2018). For instance, mutations in *fancm* helicase and *recq4* helicase or *recq4* and *figl1/flip* AAA-ATPase complex cause a 10-fold elevation in the CO rate across several genetic intervals in inbred Arabidopsis (Fernandes et al., 2018). This strategy was successfully transferred into crops with *recq4* mutant showing a significant increase in crossover frequency in rice, tomato, and pea (Mieulet et al., 2018; De Maagd et al., 2020). Surprisingly, the extra COs formed in anti-class II CO mutants are present in regions with low degree of polymorphism (Fernandes et al., 2018; Blackwell et al., 2020). In particular, *fancm* recombination phenotype seems to be sensitive to the hybrid context as it can be detected in brassica, pea, and rice but not in Arabidopsis, tomato and wheat hybrid lines. It was postulated that a high degree of polymorphism in the hybrid lines could interfere with *fancm*-dependent CO formation (Blary et al., 2018; Mieulet et al., 2018; De Maagd et al., 2020; Raz et al., 2020).

The effect of combining HEI10 over-expressor with the repression of *recq4* was tested, and the study showed a cumulative effect on CO frequency in hybrid Arabidopsis transgenic lines. However, heterochromatin recombination was not substantially increased in these lines and this strategy may have a more limited effect on crop genomes with large heterochromatin composition (Serra et al., 2018).

### Modulation of the Recombination Landscape

Meiotic recombination is not uniformly distributed along plant genomes which restricts the potential for crop improvement during breeding. In maize and barley, about 20% of all genes are located in heterochromatin, where recombination cold spot regions reside (Taagen et al., 2020), and a remodeling of the recombination landscape toward these regions could facilitate the introduction of genetic diversity. A striking negative correlation exists between CO rate, transposon content, and DNA methylation in plants (Lambing et al., 2017). In non-CG DNA methylation and H3K9me2 Arabidopsis mutant lines, the recombination landscape is altered with increased COs in centromere-proximal regions. Although DSBs are also increased, a significant deficit in DSB yield remains visible on the heterochromatin in H3K9me2-deficient mutant line, and this may be an important limiting factor for CO formation in Arabidopsis heterochromatin (Underwood et al., 2018). A direct translation of these findings to economically important crops is challenging. Epigenetic mutants in plants with larger genomes show alterations in vegetative development and fertility defects (Li et al., 2014; Tan et al., 2016; Corem et al., 2018). Alternative strategies could overcome these limitations. For example, transient silencing of epigenetic genes in reproductive tissues using virus-induced gene silencing (VIGS) could have an effect on the recombination landscape while preserving plant development.

**Figure 1:**
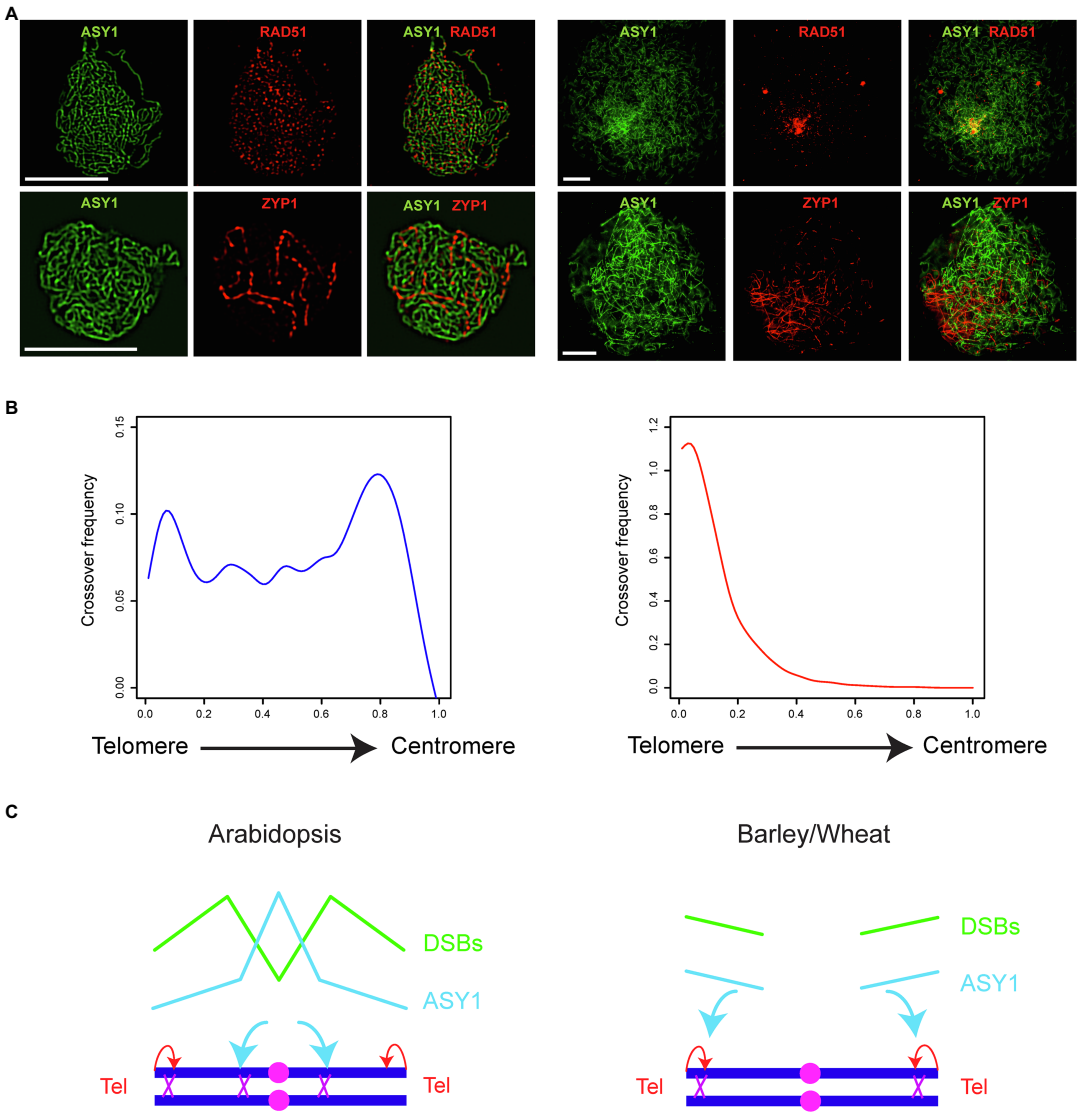
Crossover patterning in Arabidopsis and cereals. **(A)** Co-immunostaining of ASY1 (green) and RAD51 (red) at leptotene or ASY1 (green) and ZYP1 (red) at zygotene in Arabidopsis (left panel) and hexaploid bread wheat *Triticum aestivum* cv. *Cadenza* (right panel; provided by Kim Osman, University of Birmingham, United Kingdom). Scale bar = 10 μm. **(B)** Crossover frequency along an axis from telomere to centromere in Arabidopsis (blue, left panel; replotted using the CO data of all chromosomes from Serra et al., 2018) and barley (red, right panel; CO data of all chromosomes provided by Mikel Arrieta, The James Hutton Institute, United Kingdom). **(C)** Two forces influence the crossover landscape: telomere-led recombination (red arrows) and ASY1 (cyan arrows). In leptotene, axis and DSBs are formed along the chromosomes at a similar time but in distinct levels in Arabidopsis (left panel). In contrast, axis and DSBs are formed first toward the distal end of the chromosomes in barley and wheat at leptotene (right panel). This difference in the spatio-temporal formation of axis and DSBs is associated with a different landscape of crossovers between Arabidopsis and barley/wheat. Pink filled circles represent centromeres, dark blue lines represent homologous chromosomes, and purple crosses represent crossovers. The landscape of ASY1 enrichment (cyan) and DSB frequency (green) in early prophase I are represented with solid lines.

Meiotic-specific factors closely associated with recombination molecules are likely more promising targets for the control of CO landscape. For example, components of the chromosome axis are involved in the decision between inter-sister and inter-homolog recombination and Arabidopsis ASY1 and ASY3 promote CO formation (Lambing et al., 2020a). Arabidopsis ASY1 ChIP sequencing revealed that ASY1 is enriched over the centromere-proximal regions, and a gradual reduction of ASY1 is associated with a remodeling of the COs from the centromere-proximal to the distal regions (Lambing et al., 2020a). It is speculated that the distal regions are crossover prone regions due to the early homologous pairing of the telomeres while the proximal regions are crossover prone due to the enrichment of ASY1 (Armstrong et al., 2001; Lambing et al., 2020a; Figure 1C).

The CO landscape in cereals is distinct from Arabidopsis and COs are exclusively formed in distal ends of the chromosomes (Figure 1; Phillips et al., 2015; Osman et al., 2021). Moreover, the spatio-temporal formation of the chromosome axis which is observed from immunostaining of ASY1, the deposition of ZYP1 which marks synapsis between homologous chromosomes, and the formation of DSBs differ significantly between cereals and Arabidopsis (Figures 1A,C). For example, axis, synapsis, and DSB formation are initiated on the distal regions before being detected on the interstitial and centromere-proximal regions in barley and wheat (Higgins et al., 2012; Lambing et al., 2017; Desjardins et al., 2020; Osman et al., 2021). In contrast, no polarization of axis formation or DSB formation is detected in Arabidopsis (Lambing et al., 2017; Figure 1A). It is conceivable that COs are exclusively distal in cereals because the distal regions experience first the formation of DSBs and the pro-CO activity of ASY1 (Figures 1B,C). In this context, it is important to remodel ASY1 ons the chromosomes to achieve a remodeling of the CO landscape in cereals. Indeed, this can be achieved by increasing the temperature in barley (Higgins et al., 2012). The change of temperature reduces the polarization of axis formation, and ASY1 is detected more evenly on the chromosomes which is associated with an elevation of interstitial and centromere-proximal chiasmata (Higgins et al., 2012). However, this strategy may not be applicable to every crops, as seen in the observation that wheat recombination is only slightly and locally altered at high temperature (Coulton et al., 2020).

### Targeted Recombination

**Figure 2:**
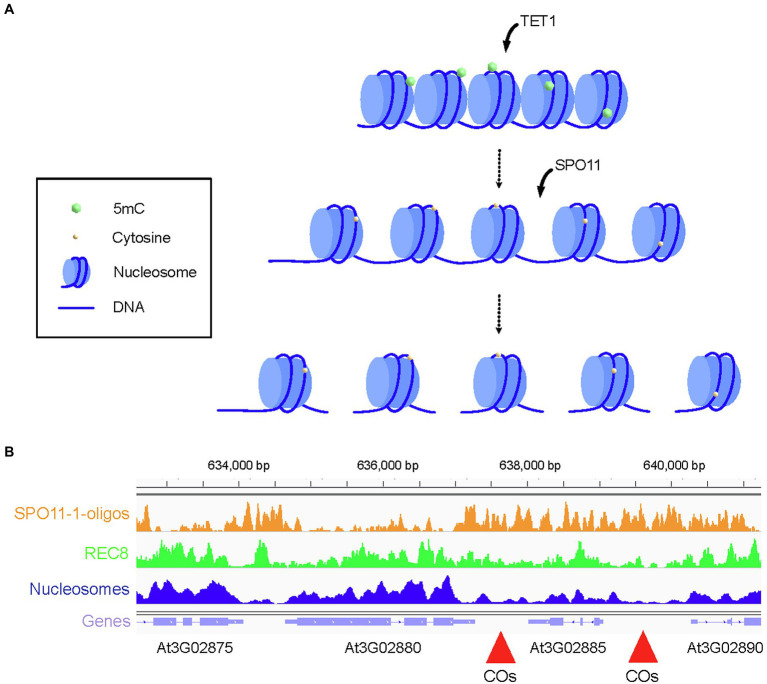
Strategies to remodel the crossover rate locally. **(A)** Representation of a cold spot region enriched in nucleosome and silencing epigenetic marks, such as DNA methylation in all three contexts (CG, CHG, and CHH). Chromatin is methylated on 5mCs. Targeted recruitment of TET1 catalyzes the removal of silencing epigenetic marks and decompaction of chromatin while the targeted recruitment of SPO11 catalyzes formation of DSBs. Meiotic DSBs are repaired by the homologous recombination pathway leading to the formation of NCO, NCO with gene conversion, or CO. **(B)** Genome browser view of the crossover hotspot *3a* on chromosome 3 of Arabidopsis. SPO11-1-oligo (orange), REC8 ChIP-seq (green), and nucleosome (MNase, dark blue) profiles are shown alongside the gene organization (purple). Regions with high crossover rate are indicated with red arrowheads. Coordinates on the chromosome 3 are shown above the genome browser view.

Targeting recombination is potentially a preferred strategy compared to a genome-wide change in CO frequency, because it allows precise positioning of recombination events on the genome. This could be achieved by targeting recombination proteins to a specific locus or to locally alter the epigenome. DSBs are generally enriched in promoters, introns, and terminators of genes (Choi et al., 2018) and are depleted in exonic regions that are enriched in nucleosome and axis REC8 cohesin (Figure 2B; Choi et al., 2018; Lambing et al., 2020b). Electron microscopy studies show that the chromosome axis forms an electron dense structure (Kleckner, 2006) and the compact structure of the axis could inherently prevent DSB formation even if SPO11 is targeted to this region. Therefore, a fine analysis of the chromatin landscape appears important for the design of targeted recombination to maximize the efficiency of DSB formation.

CO cold spots have generally low DSB frequency and are enriched in nucleosome density, DNA methylation, and epigenetic silencing marks (Yelina et al., 2015; Choi et al., 2018; Underwood et al., 2018). Loss of DNA methylation is associated with a gain of DSBs in Arabidopsis (Choi et al., 2018; Underwood et al., 2018) and represents an interesting strategy for targeted recombination. In Arabidopsis, DNA is actively demethylated by ROS1 and related glycosylase enzymes through a base excision-repair process (Gong et al., 2002; Penterman et al., 2007; Zhu et al., 2007; Zhang and Zhu, 2012). An alternative pathway dependent on Ten-eleven translocation methylcytosine dioxygenase 1 (TET1) exists in human that biochemically removes DNA methylation. TET1 catalyzes the oxidation of 5-methylcytosine (5mC) to 5-hydroxymethylcytosine which is the initial step for DNA demethylation (Wu and Zhang, 2017). Fusion of human TET1 to an artificial zing finger or to CRISPR/dCas9 effectively demethylates DNA at targeted loci in Arabidopsis (Figure 2A; Gallego-Bartolome et al., 2018). This method could in theory be used in conjunction with CRISPR/dCAS9 fused with SPO11 or a component of the DSB machinery to promote DSB formation in an otherwise cold region (Figure 2A).

The recruitment of SPO11 protein to a specific locus does not necessary ensure the formation of a DSB (even less so a CO-prone DSB). To form a DSB, SPO11 requires not only to be part of a protein complex but also to be functionally active (Robine et al., 2007; Sarno et al., 2017). Studies from budding yeast revealed that not every locus bound by SPO11-GAL4 is proficient to form DSBs, and the establishment of a DSB is determined by local factors (Robine et al., 2007; Sarno et al., 2017). In plants, a recent study suggests that expression of a MTOPVIB-dCas9 fusion protein to induce targeted meiotic DSB within a CO hotspot located in a subtelomeric region of chromosome 3 is not sufficient to affect CO frequency (Yelina et al., 2021). In budding yeast, it is estimated that around 40% of the DSBs are converted to COs (Mancera et al., 2008). In contrasts, in Arabidopsis (Ferdous et al., 2012), maize (Sidhu et al., 2015), and barley (Higgins et al., 2012) only about 2–5% of the DSB rate accounts for the total CO number and it is likely that the targeted DSBs will convert to COs at low frequency. In addition, a budding yeast study showed that expression of SPO11-GAL4 in a *spo11* null mutant forms DSBs at GAL4 sites but also at natural sites (Pecina et al., 2002). If conserved in plants, this propensity of SPO11 may further reduce the targeted effect. Moreover, barley and wheat chromosome axes initiate first in distal regions in early prophase (Higgins et al., 2012; Osman et al., 2021). It is unknown whether SPO11 is functionally active to form a DSB without a chromosome axis when recruited in centromere-proximal regions at an early stage of meiosis.

The induction of targeted recombination can have undesirable effects on recombination elsewhere in the genome. For example, the formation of DSBs at targeted sites inhibits the formation of DSBs in adjacent natural sites in budding yeast (Robine et al., 2007). Moreover, if the targeted DSB is converted to a CO this will inhibit the formation of a second CO in the adjacent regions *via* a phenomenon called CO interference (Berchowitz and Copenhaver, 2010). In Arabidopsis, the effect of CO interference is detected over 8–10 Mb of DNA and the formation of a targeted CO will likely remodel the recombination landscape on that chromosome (Serra et al., 2018). Unlike COs, gene conversions have relatively short length (<2 kb) and are detected in most parts of the genome including across centromeric regions in plants (Shi et al., 2010). This type of recombination events is interesting because it only modifies the DNA sequence locally and does not seem to be under the same controls as COs (Shi et al., 2010). Moreover, targeted gene conversion events are unlikely to modify the broad CO landscape (Berchowitz and Copenhaver, 2010). This outcome is particularly interesting for plant breeding where targeted recombination is required to increase allelic diversity locally, such as in heterochromatic regions.

## Chromosome Engineering to Influence Meiotic Recombination

### Chromosome Structure and Crossovers

Chromosome structure is also a strong determinant of CO formation and localization (Rowan et al., 2019). Chromosomal rearrangements, such as inversions or translocations, usually suppress recombination, and this is particularly challenging for breeding since they may inhibit the transfer of important traits between different plant cultivars. Indeed, a number of inversions and translocations can be detected by comparing the genomic sequences between accessions (Zapata et al., 2016). Recently, somatic chromosomal engineering using CRISPR/Cas9 has proven useful for restoring recombination in naturally rearranged chromosomal regions in Arabidopsis (Schmidt et al., 2020). A particularly well-known case of inversion in Arabidopsis is the heterochromatic knob on chromosome 4 (Fransz et al., 2000). When an Arabidopsis accession carrying this inversion is crossed with an accession without inverted knob, CO formation within the entire rearranged region is suppressed (Schmidt et al., 2020). The authors developed an egg cell-specific expression system of the Cas9 nuclease that allows rearranging the structure of plant chromosomes in a targeted and heritable manner (Beying et al., 2020; Schmidt et al., 2020). Remarkably, reversion of the 1.1 Mb heterochromatic knob on chromosome 4 fully restored CO formation in this region (Schmidt et al., 2020). This is a particularly promising achievement for breeding given that many crop plants have experienced substantial chromosomal rearrangements that strongly affect CO formation.

### Effect of Ploidy Manipulation on Crossovers

Interestingly, a link between increased ploidy level and crossover formation has been demonstrated in a number of plants (reviewed in Pele et al., 2017). For instance, in Arabidopsis, analyses of CO formation in one genetic interval show that both male and female CO frequencies are significantly higher in newly formed auto- and allopolyploids compared to their diploid progenitors (Pecinka et al., 2011). Additionally, studies in Brassica demonstrated that Brassica allotriploid hybrids exhibit a significant crossover increase compare to their progenitors (Leflon et al., 2010; Suay et al., 2014; Pele et al., 2017). This increase occurs genome wide and affects both male and female meiosis, although stronger increase is observed in female and is associated with a significant remodeling of the CO landscape with the presence of COs in the vicinity of centromeres. Remarkably, this increase is also accompanied by a strong decrease in CO interference (Suay et al., 2014; Pele et al., 2017). Although the underlying mechanism remains to be demonstrated, it appears that the link between ploidy level and CO increase is associated with genetic content. Indeed, further work in Brassica has shown that the addition of one specific chromosome (C genome chromosome 9) is sufficient to increase CO in polyploid hybrids while addition of other chromosomes had no effect (Suay et al., 2014). Altogether, these results suggest that manipulating ploidy level and/or chromosome composition may be a promising alternative for plant breeders to modulate CO formation and ultimately increase genetic diversity of crop plants.

## How can we Remodel Meiosis for Crop Improvement?

The manipulation of meiotic recombination gives the breeders a tool to create a new and desirable allele of gene that could be incorporated to a germ line and, unlike the product of mitotic recombination, this trait will be carried to the whole plant as it develops. However, such trait can be removed/modified as meiotic recombination continuously occurs in the following generations. Moreover, the process of meiosis maintains the ploidy of the progeny and limits cross-breeding between accessions or related species containing different ploidy. To overcome these constraints for crop breeding, the meiotic division processes could be engineered to adapt the need of a breeding program.

### Diploid Gametes

**Figure 3:**
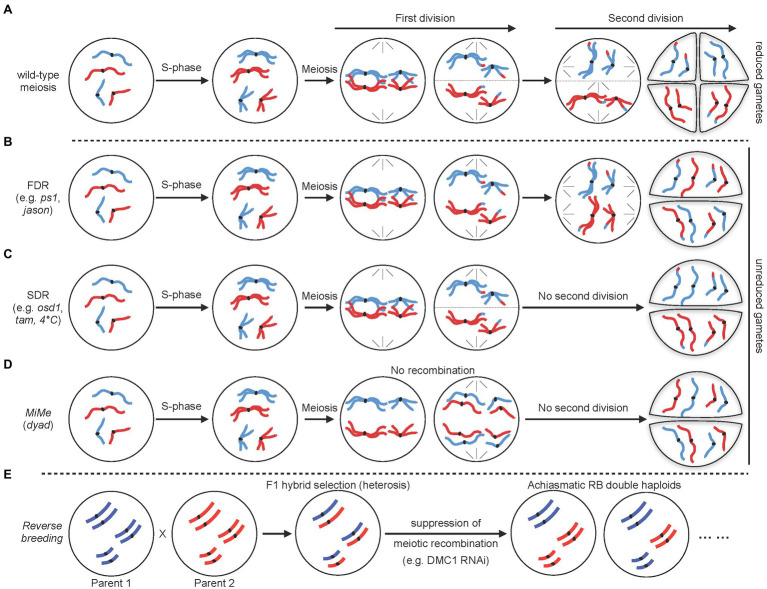
Schematic representation of different strategies to remodel the meiotic division. **(A)** Normal meiotic division resulting in haploid (n) gametes. **(B,C)** Non-reductional meiotic divisions resulting in unreduced (2n) meiotic products. First division restitution (FDR; **B**) and second division restitution (SDR; **C**) lead to different levels of heterozygosity (see text for details). **(D)** Schematic diagram of apomeiosis obtained through MiMe strategy. **(E)** Reverse breeding strategy. For simplification, in each case, a diploid cell with only two chromosome pairs is shown with maternal and paternal chromosomes in red and blue, respectively.

An important application for remodeling the meiotic division process is to allow formation of unreduced gametes (Figure 3). Indeed, a major function of meiosis is to reduce the chromosome complement by half with two successive divisions following a single round of DNA replication. Consequently, circumvention of one division allows formation of unreduced gametes which have proved useful for breeding. Specifically, unreduced gametes are used by breeders to engineer sexual polyploidization (Brownfield and Kohler, 2011; Crismani and Mercier, 2012; De Storme and Geelen, 2013; Ronceret and Vielle-Calzada, 2015). They can facilitate crosses between plants with different ploidy levels or to be utilized to create new polyploid species exhibiting increased genetic diversity and hybrid vigor. It has long been considered that formation of diploid gametes is, at least in part, genetically controlled. Accordingly, a number of mutants producing diploid gametes have been identified in various plants (for reviews see Brownfield and Kohler, 2011; Crismani and Mercier, 2012; De Storme and Geelen, 2013; Ronceret and Vielle-Calzada, 2015). These mutants are usually classified as first division restitution (FDR) or second division restitution (SDR) depending on whether the mutations affect the first or second division, respectively (Figures 3A–C). In Arabidopsis, notable examples of these are mutations in *parallel-spindle 1* or *Jason* that both lead to FDR through disturbance of spindle orientation and positioning (D’Erfurth et al., 2008; De Storme and Geelen, 2011). On the contrary, SDR has been obtained by mutating genes controlling entry into second division, such as OMISSION OF DIVISION 1 (*OSD1*) a key regulator of the anaphase promoting complex, or the cyclin *CYCA1;2/*TARDY ASYNCHRONOUS MEIOSIS (*TAM1*; D’Erfurth et al., 2009, 2010). A key difference between FDR and SDR is that they lead to different genetic outcomes. FDR-influenced chromosomes are non-sister chromatids, and therefore, FDR is often considered to produce unreduced gametes with enriched heterozygous marker genotypes (from centromeres to first crossover sites). On the contrary, in SDR, affected chromosomes are sister chromatids (second division not occurring) and the unreduced gametes exhibit homozygous marker genotypes from the centromeres to the first crossover site. Hence, it is important to take into account the desired level of heterozygosity when considering FDR or SDR for a breeding strategy. Interestingly, diploid gametes may also be obtained by applying external stimuli. For example, a high number of diploid gametes are produced when haploid Arabidopsis plants are treated with a 4°C cold shock for several hours during flowering stage and this process primarily undergoes SDR (De Storme and Geelen, 2011). Whether this strategy is effective in crops and if other stimuli may trigger similar effects are not yet known. Nevertheless, such approach may be highly relevant for breeding since it would be classified as non-transgenic.

### Apomeiosis

Both FDR and SDR result in unreduced gametes which contains chromosomes that were recombined *via* meiotic recombination. However, regarding to breeding strategies, unreduced gametes that have retained parental genome are more useful. Apomixis, in particular, is a form of asexual reproduction allowing clonal reproduction through seeds (Spillane et al., 2004). Apomixis produces progenies that are genetically identical to the maternal genome. This is especially beneficial on the breeding of hybrid varieties since it allows fixation of an elite hybrid genome and its clonal propagation through seeds. Although apomixis naturally occurs in a number of angiosperms, it is absent in most important crops (Spillane et al., 2004). Its success relies on the circumvention of both meiosis and fertilization. A cornerstone of apomixis is thus apomeiosis, a deregulated form of meiosis resulting in a mitotic-like division that prevents recombination and ploidy reduction. Several single mutants disrupting the meiotic process and leading to apomeiosis have been identified in Arabidopsis, rice, and maize (Ronceret and Vielle-Calzada, 2015; Figure 3D). However, these mutants are usually sterile and form apomeiotic gametes at an extremely low frequency. The best example of these is the Arabidopsis *DYAD/SWITCH1* gene, a regulator of meiotic chromosome organizations essential for meiotic entry (Ravi et al., 2008). Artificial apomeiosis has been successfully achieved through mutation of this single gene (Ravi et al., 2008). In the *dyad* allele, alteration of the meiotic process into a mitotic-like division leads to the formation of unreduced female gametes that retain parental heterozygosity, representative of apomeiosis. However, although this appeared promising at first sight, it is hardly amenable to crops since *dyad* plants are nearly sterile and less than 1% of the *dyad* ovules generate viable gametes (Ravi et al., 2008).

Rather than mutating a single gene, a major success in engineering apomeiosis was later obtained by combining several mutations that disrupt the key steps of the meiotic division (D’Erfurth et al., 2009; Figure 3D). This was accomplished by simultaneously disrupting three key steps of meiosis: (1) bypassing of the second meiotic division to allow production of functional diploid gametes; this can be achieved through mutating and removing the function of the key regulator *OSD1*, (2) suppression of meiotic recombination to prevent formation of recombined gametes. This can be achieved through mutation of genes involved in meiotic DSB formation. For example, using a null allele of *SPO11-1* to eliminate the initiation of meiotic DSB formation, and (3) allowing segregation of sister chromatids at the first division through loss-of-function of the meiotic-specific cohesin REC8. In Arabidopsis, plants with this genotype undergo meiosis that is replaced by mitosis and they are called *MiMe* (for Mitosis instead of Meiosis). This remodeling of meiosis gives rise to functional diploid gametes with genetically identical genomes (D’Erfurth et al., 2009; Figure 3D). The practicability of this technology was further demonstrated by alternatively targeting other genes disrupting the key steps of meiosis. For instance, the use of *osd1* mutation to bypass the second meiotic segregation has been successfully replaced by mutation of the cyclin *CYCA1; 2/TAM1* or use of a *tdm1* dominant mutation (D’Erfurth et al., 2010; Cifuentes et al., 2016). Similarly, suppression of meiotic recombination can be obtained by mutating various components of the meiotic DSB complex (e.g., *PRD1*, *PRD2*, or *PRD3*; Mieulet et al., 2016). This artificial engineering of apomeiosis is a particularly ground-breaking achievement since *MiMe* plants are fertile and produce near wild-type levels of viable apomeiotic gametes. Remarkably, this technology has also recently been translated to rice (Mieulet et al., 2016). Through mutation of rice *OSD1*, *PAIR1* (rice homolog of *PRD3*), and *REC8*, the authors could reproduce the *MiMe* genotype and, importantly, showed that rice *MiMe* plants remained fertile. Altogether these data demonstrate the potential of the *MiMe* technology for engineering apomeiosis in plants. Yet, it remains unclear whether this technology is applicable to other crops and, in particular, polyploid species, such as bread wheat. Another obstacle of this technology is that since gametes are diploid and normal fertilization continues to occur, ploidy is expected to double at each generation. To overcome this problem, *MiMe* technology has been combined with genome elimination strategies that allow removal of a complete set of chromosome from one parent after fertilization (Ishii et al., 2016; Jacquier et al., 2020). Such genome elimination is usually accomplished by using haploid inducer lines which can be obtained through manipulation of the centromeric histone 3 variant (CENH3; Ravi et al., 2008; Marimuthu et al., 2011), or the *MATRILINEAL*/*NOT-LIKE DAD*/*PHOSPHOLIPASE A1* gene (Wang et al., 2019a). Haploid inducer lines do not directly affect meiosis and will thus not be described here. For detailed description of haploid inducer lines and genome elimination, readers are directed to several excellent recent reviews (Ishii et al., 2016; Jacquier et al., 2020). Alternatively, creation of haploid plants can also be obtained by misexpression in egg cell of the *BABY BOOM 1* (*BBM1*) gene (Khanday et al., 2019). *BBM1* is a male-expressed gene essential to initiate embryogenesis after fertilization and misexpression of *BBM1* in egg cell is sufficient to trigger parthenogenesis and the production of haploid plants (Khanday et al., 2019). Remarkably, combining one of these strategies with MiMe technology has allowed engineering clonal reproduction in both Arabidopsis and rice (Marimuthu et al., 2011; Khanday et al., 2019; Wang et al., 2019a; Khanday and Sundaresan, 2021). Yet, frequencies of clonal embryo remain low (haploid inducer lines are not fully penetrant) and overall seed production is also strongly decreased. This means that broad implementation of apomixis in a sustainable way in crops will require further research to unravel new factors and mechanisms controlling apomeiosis and haploid induction. However, to achieve this will also require a better understanding of the interplay between these two components of apomixis.

### Reverse Breeding

Heterozygous hybrids have the tendency to outperform their homozygous parents in fitness (Chen, 2010; Labroo et al., 2021). This phenomenon, known as hybrid vigor or heterosis, is widely used by breeders to produce elite varieties with improved field quality. Creation of these elite heterozygous hybrids is achieved by crossing two selected homozygous parents. However, such favorable hybrids cannot be stably maintained because allele combinations are reshuffled by genetic recombination during meiosis before being transmitted to the progeny. This means that offspring lose the heterosis effect and breeders must continuously recreate favorable hybrids. Different strategies have been proposed to preserve transmission of heterozygous genotypes. Reverse breeding, which is an alternative to apomixis, has emerged as a promising strategy to fix hybrid genomes (Dirks et al., 2009; Figure 3E). Reverse breeding generates homozygous parental lines from a heterozygous hybrid. When applied to hybrids with known parents, the approach can also be used to generate chromosome substitution lines, in which the chromosome of one line is replaced by the chromosome of another line (Dirks et al., 2009). The method relies on suppression of meiotic crossovers in the hybrid followed by the production of doubled haploids from non-recombinant gametes (Dirks et al., 2009; Figure 3E). The practicability of the method was originally demonstrated in Arabidopsis by silencing the meiotic-specific recombinase DMC1 using RNA interference (Wijnker et al., 2012, 2014). Non-recombinant gametes were converted into haploid adult plants using centromere-mediated genome elimination and fertile diploids (double haploids) and were eventually obtained by self-pollination of those haploids (Wijnker et al., 2012). The main limitation of the method lies in the fact that suppression of meiotic recombination leads to achiasmatic chromosomes which segregate randomly. Production of balanced non-recombinant gametes thus relies on fortuitous balanced segregation, whose frequency strongly depends on chromosome number. The method is thus limited to species with low chromosome number (less than 12; Dirks et al., 2009). An alternative to the solution would be to reduce, but not completely suppress, CO formation. Having one or a few CO would still lead to low production of CO-free gametes but will also increase the production of gametes with low CO numbers, which would prove useful for reverse breeding. This strategy was recently validated by downregulating Arabidopsis *MSH5* gene expression through VIGS (Calvo-Baltanas et al., 2020). Furthermore, VIGS has the additional advantage of allowing transient downregulation and thus avoids integration of a stable transgene in the genome, which is a strong concern for breeders. Altogether, these data suggest that reverse breeding could be effectively applied to many crops. However, unlike apomixis, this strategy has not yet been demonstrated in crops.

## Concluding Remarks

Several novel insights on meiosis have emerged in recent years and form a framework to develop innovative technologies to accelerate pre-breeding strategies. However, most of our understanding of meiosis is based on Arabidopsis research and it is of particular importance to pivot toward the translational potential of these discoveries and the study of other plant species. It is likely that future studies will identify significant differences in the regulation of meiosis between Arabidopsis and crops which may occlude a direct transfer of certain strategies between plants. Comparative studies of meiosis across a broad range of species will address this gap in our knowledge and have the potential to identify new functional pathways and to provide a deeper understanding of the evolution of meiotic gene function.

## Author Contributions

All authors listed have made a substantial, direct and intellectual contribution to the work, and approved it for publication.

### Conflict of Interest

The authors declare that the research was conducted in the absence of any commercial or financial relationships that could be construed as a potential conflict of interest.

The reviewer SH declared a past co-authorship with one of the authors CL to the handling editor.
